# Diurnal Variation in Visual Simple Reaction Time between and within Genders in Young Adults: An Exploratory, Comparative, Pilot Study

**DOI:** 10.1155/2021/6695532

**Published:** 2021-01-22

**Authors:** Hanumantha S, Ashwin Kamath, Rajeshwari Shastry

**Affiliations:** ^1^Kasturba Medical College, Mangalore, Manipal Academy of Higher Education, Manipal, Karnataka, India; ^2^Department of Pharmacology, Kasturba Medical College, Mangalore, Manipal Academy of Higher Education, Manipal, Karnataka, India

## Abstract

Simple reaction time (SRT) is the minimum time required to respond to a stimulus; it is a measure of processing speed. Our study aimed to determine the variation in visual SRT with time among individuals of the same gender and between genders. We carried out a prospective, parallel group, pilot study involving ten male and ten female medical students aged 18–25 years. After obtaining written informed consent, the participants were familiarized with the procedures, and each completed a single practice session of a computerized visual SRT which was administered using Psychology Experiment Building Language Version 2.0 software. On a predetermined day, the participants completed the exercise at 10 a.m., 1 p.m., and 5 p.m. The results showed no statistically significant difference in SRT based on time of day between genders (*χ*^2^(2) = 4.300, *p*=0.116) as well as within gender (males (*χ*^2^(2) = 0.600, *p*=0.741); females (*χ*^2^(2) = 5.000, *p*=0.082). Our study showed that visual SRT does not change significantly at different times of the day and within and between genders. Intraindividual variations in visual SRT can mask the presence of a small but significant difference; hence, further studies are warranted.

## 1. Introduction

Simple reaction time (SRT) is the minimum time required to respond to a stimulus; it is a measure of processing speed [1]. Earlier studies have reported visual SRTs ranging from 231 ms to 397 ms [1, 2]. SRT depends on perception (hearing, seeing, and feeling of a stimulus), processing (focus and understanding the information), and response (motor agility) [3]. It also depends on the type and complexity of the stimulus, the stimulated sensory modality, familiarity, preparation, expectations, and state of the individual [3–5]. Various aspects of SRT have been addressed by earlier research, such as the effect of age, sex, and body mass index on visual and auditory SRT and latency of reaction time [1, 6], time course of corticospinal excitability during SRT task, [7] and validation of the reaction time [5].

A number of studies have reported a faster reaction time in males compared to females [8–10]. However, there are studies which have found either no difference or absence of difference based on the stimulus presented [1, 11]. In addition, intraindividual variation in SRT has been reported across the lifespan [9]. There are no adequate reports regarding the circadian variation in SRT and whether this is different based on gender.

The current study intended to explore the changes, if any, in visual SRT with time among individuals of the same gender and between genders using a simple computer-based psychometric task. The Psychology Experiment Building Language (PEBL) open-source software consists of a battery of psychological/psychometric tests that help in rapid, uniform, and standardized administration of the tests to participants [12]. We used the PEBL simple reaction time task to measure the visual SRT in this study. The use of a computerized psychometric test, which is rapid and easy to administer, in a group of male and female participants would help us determine if there is any variation in the visual SRT with time, among and between genders.

## 2. Materials and Methods

We conducted a prospective, parallel group, pilot study among 10 male and female undergraduate medical students aged between 18 and 25 years in the Clinical Pharmacology Lab of Kasturba Medical College, Mangalore, India. The study was initiated after receiving approval from the institutional ethics committee and registration of the study protocol in the Clinical Trial Registry of India (CTRI/2019/06/019848). The study was conducted in accordance with the Ethical Guidelines for Biomedical Research on Human Subjects (Indian Council of Medical Research) and the Declaration of Helsinki. The sample size was determined based on feasibility. Sixth-semester medical students were invited to participate in the study. The first twenty consenting eligible participants (10 males and 10 females) were enrolled in the study. The inclusion criteria were as follows: healthy subjects of either gender, 18 to 25 years of age, willing to provide informed consent, and willing to follow the study procedures. Exclusion criteria included presence of any acute/chronic illness at the time of participation, history of intake of any medication within a week prior to participation in the study, or unlikely to comply with the study requirements as per the opinion of the investigator.

After obtaining written informed consent, 10 male and 10 female participants were familiarized with the test procedures, and each of them completed a single practice session of the computerized visual SRT. The participants were instructed not to consume stimulant drinks on the day of the experiment. None of the participants had history of alcohol or tobacco use or smoking. Then, on a predetermined day, the participants completed the exercise at three different times of the day, i.e., at 10 a.m., 1 p.m., and 5 p.m. We chose these three day-time time points to explore the potential changes in visual SRT based on the findings of earlier research which studied various cognitive parameters and found evidence for a circadian change in these parameters during the wake time [13–15]. The 10 a.m. SRT task was performed >1 hour after food intake; the 1 p.m. and 5 p.m. SRT tasks were performed ≥3 hours after food intake. All the participants belonged to the same academic batch and performed the same academic activities on the scheduled day. The visual SRT was assessed using the PEBL Version 2.0 software, which was preinstalled in laptops before the session. Each participant completed a single run of 10 trials of the test, and any doubt regarding the study procedure was clarified. Following the trial session, the participant completed a test run of 50 trials at each time point (three time points on the scheduled day). The mean reaction time for the 50 trials during each run was used for the statistical analysis. Anticipations (reaction time <150 milliseconds) and delayed responses (reaction time >3000 milliseconds) were not included in the calculation of the mean reaction time; these were the default limits already set in the PEBL software.

## 3. Statistical Analysis

The normality of data distribution was assessed using the Shapiro–Wilk test. Since the data were not normally distributed (*p* < 0.05), nonparametric repeated measures analysis of variance (Friedman test) was used to determine the presence of any difference in the reaction time at different time points; we also analyzed the data separately for each gender to determine the presence of any within-gender change in SRT with time. A *p* value <0.05 was considered statistically significant. The comparison of reaction times at individual time points between genders was performed using nonparametric one-way analysis of variance (Kruskal–Wallis test). Bonferroni's correction was used to adjust for multiple comparisons, which yielded a *p* value of 0.017. Data analysis was performed using Statistical Package for Social Sciences, Version 11.5 (Chicago, IL, USA).

## 4. Results

**Figure 1:**
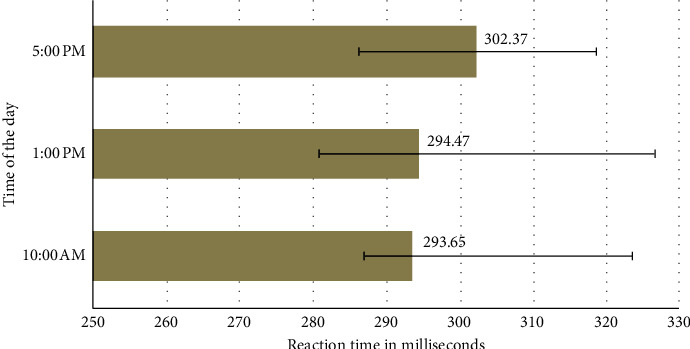
Visual simple reaction time of study participants at different times of the day. The data shown are the median reaction time; the horizontal error bars represent the 25th and 75th percentiles.

Ten male and ten female participants completed the study. The median visual reaction times of the entire study sample at three different times of the day are shown in Figure 1. There was no statistically significant difference in SRT based on time of the day (*χ*^2^(2) = 4.300, *p*=0.116). The same was also true when the results were analyzed separately in males (*χ*^2^(2) = 0.600, *p*=0.741) and females (*χ*^2^(2) = 5.000, *p*=0.082).

**Figure 2:**
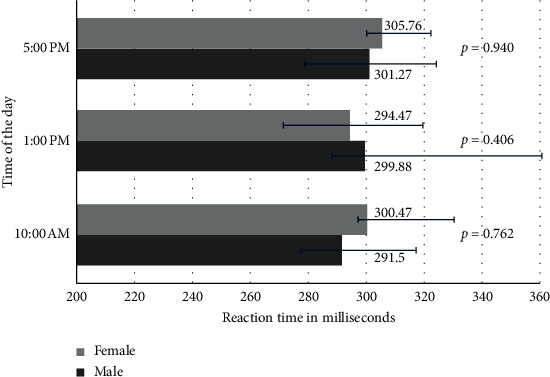
Comparison of the visual simple reaction time of study participants at different times of the day based on gender. The data shown are the median reaction time; the horizontal error bars represent the 25th and 75th percentiles.

To determine the presence of any difference in the SRT between genders, we compared the SRT data at individual time points between the genders. No significant difference in the SRT was seen between males and females at any of the time points (Figure 2). The median number of anticipations in males and females was 1 (0–2) and 3 (1–5), respectively (*p*=0.105). No delayed responses were seen in either group.

**Figure 3:**
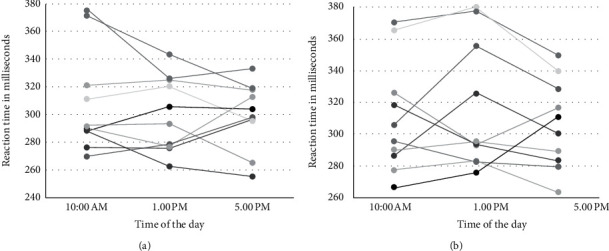
Changes in the visual simple reaction time of study participants (shown using different color lines) at different times of the day in (a) male participants and (b) female participants.

Figure 3 shows the variation in the SRT in male and female participants. As can be seen, there is no definite pattern noted regarding the changes in the SRT.

## 5. Discussion

Our study measured the visual SRT in ten male and ten female participants at three different time points of the day using a computer-based test. The study results did not show any statistically significant difference in SRT at different time points of the day within and between genders.

The absence of any difference in the visual SRT in the overall study sample is in contrast to the findings of some of the earlier studies. Pomplun et al. [16] studied the circadian phase effect, influence of wake time, and chronic sleep restriction effects on tasks requiring visual working memory and attentional control using two comparative visual search tasks in 12 healthy young adults, 6 males, and 6 females; they found that the time of the day, as well as the duration of remaining awake, significantly affected the response time. However, no significant effect was seen on the accuracy of performance of the task. Horowitz et al. [17] also studied the influence of wakeful time period and circadian rhythm on visual search using spatial configuration and conjugation search tasks; increased wakeful time and adverse circadian phases prolonged the reaction time. However, both the above studies involved testing under extreme conditions (prolonged wakefulness), unlike our study where the tests were conducted during the day with a gap of about 4 hours between each time point. The significant differences seen in these studies were in response to chronic sleep restriction. Regarding the influence of the circadian phase in these studies, the performance was found to be poorer at a time just after waking [16]. In our study, the session began after the morning classes for the participants; there was no sleep restriction, and the participants were well active during the session. In addition, the tests used in the earlier studies were relatively more complex, involving more complex mental processes in the completion of the task than the simple visual SRT test used in the current study. For example, in the study by Pomplun et al. [16], the tasks involved copying and mirroring tasks, the later requiring mental image transformation. In the study by Horowitz et al. [17], the tasks involved finding a number among a group of incongruent numbers and finding vertical lines of a particular color among horizontal and vertical lines of other colors. This may partly explain the absence of any significant difference in the reaction time among our study participants.

With regard to gender, earlier studies have shown conflicting results. Our study showed an absence of gender difference in the visual SRT. This is in agreement with the findings of Woods et al. [1], who used a simple visual search task in 1469 participants similar to that used in our study, Kandil et al. [11], and the observations of Silverman [18]. Regarding the small gender differences that have been reported in earlier studies [8–10], the differences can, at least partly, be attributed to age differences and the level of physical activity [9]. Better motor responses would be associated with better performance in tasks that involve, for example, pressing a button. A study by Jain et al. [8] found that medical students who exercised regularly had faster reaction times compared with those who had a sedentary lifestyle. Dykiert et al. [9] found a significant gender difference in SRT in adults but not in children; they propose that the difference is likely due to the influence of hormones on the brain; however, no gender difference was seen in the choice reaction time.

Our study has limitations. All time points were during the day time and, hence, did not entirely cover the circadian influence. The sample size was small and was not adequately powered to detect small differences between the groups. However, many earlier studies that have shown differences have also involved small sample sizes. Our study did not test the influence of fatigue or prolonged tasks on the reaction time since the task at each time point was completed within about 6 minutes.

## 6. Conclusions

Our study showed that visual SRT does not change significantly at different times of the day, within and between genders. Considering the large variations seen in the visual SRT within individuals, such variations can introduce significant noise in the data and can lead to both false-positive and false-negative results; hence, studies using larger samples are required to detect small significant differences, if any.

## Data Availability

The data that support the findings of this study are available from the corresponding author upon reasonable request.
